# Telomerase in Cancer: Function, Regulation, and Clinical Translation

**DOI:** 10.3390/cancers14030808

**Published:** 2022-02-05

**Authors:** Nathaniel J. Robinson, William P. Schiemann

**Affiliations:** Case Comprehensive Cancer Center, Case Western Reserve University, Cleveland, OH 44106, USA; njrobins@uab.edu

**Keywords:** telomeres, telomerase, TERT, TR, extratelomeric, targeted therapy, treatment resistance

## Abstract

**Simple Summary:**

Cells undergoing malignant transformation must circumvent replicative senescence and eventual cell death associated with progressive telomere shortening that occurs through successive cell division. To do so, malignant cells reactivate telomerase to extend their telomeres and achieve cellular immortality, which is a “Hallmark of Cancer”. Here we review the telomere-dependent and -independent functions of telomerase in cancer, as well as its potential as a biomarker and therapeutic target to diagnose and treat cancer patients.

**Abstract:**

During the process of malignant transformation, cells undergo a series of genetic, epigenetic, and phenotypic alterations, including the acquisition and propagation of genomic aberrations that impart survival and proliferative advantages. These changes are mediated in part by the induction of replicative immortality that is accompanied by active telomere elongation. Indeed, telomeres undergo dynamic changes to their lengths and higher-order structures throughout tumor formation and progression, processes overseen in most cancers by telomerase. Telomerase is a multimeric enzyme whose function is exquisitely regulated through diverse transcriptional, post-transcriptional, and post-translational mechanisms to facilitate telomere extension. In turn, telomerase function depends not only on its core components, but also on a suite of binding partners, transcription factors, and intra- and extracellular signaling effectors. Additionally, telomerase exhibits telomere-independent regulation of cancer cell growth by participating directly in cellular metabolism, signal transduction, and the regulation of gene expression in ways that are critical for tumorigenesis. In this review, we summarize the complex mechanisms underlying telomere maintenance, with a particular focus on both the telomeric and extratelomeric functions of telomerase. We also explore the clinical utility of telomeres and telomerase in the diagnosis, prognosis, and development of targeted therapies for primary, metastatic, and recurrent cancers.

## 1. Introduction

In higher eukaryotes, somatic cells undergo a finite number of divisions before entering senescence and ultimately undergoing apoptosis, thereby preventing the generation and accumulation of pathologic genetic anomalies [1]. However, cells capable of circumventing these defenses typically exhibit enhanced proliferative abilities, together with heightened capacities to evade growth suppressing and cell death signals. These features constitute several of the hallmarks of cancer, which also include mechanisms governing the manipulation of local immune and angiogenic microenvironments, as well as those promoting the metastatic dissemination of cancer cells [2]. Thus, genomic instability can be viewed as a wellspring for many of the remaining cancer hallmarks, with the attainment of replicative immortality serving as a necessary condition for the propagation of tumor-promoting genomic aberrations. Telomeres, the terminal structures of linear chromosomes, provide a substrate for cellular aging and function as essential determinants of replicative potential [3,4]. Consequently, aberrant telomere homeostasis imparts cells with replicative immortality [5]. More broadly, telomeres and their associated factors have been implicated in regulating nearly all of the tumorigenic features of cancer, including genome instability [6,7], sustaining proliferative signaling [8,9], evading growth suppressors [10], resisting cell death [11], inducing angiogenesis [12,13] and immune modulation [14], and activating invasion and metastasis [15]. Importantly, the functional versatility of telomeres is determined by their structural dynamics and the regulatory mechanisms that exist to maintain them.

Telomeres are nucleoprotein structures composed of tandem DNA repeats ((TTAGGG)_n_, average length of ~10 kb [16] in humans) and a core six-member protein complex known as shelterin [17,18]. Shelterin-bound telomeres provide parallel solutions to two potentially catastrophic problems posed by the packaging of genetic information into linear, discontinuously-replicated chromosomes: (i) the end-replication problem, which describes the iterative loss of genetic information secondary to asymmetric replication of DNA strands [19,20]; and (ii) the end-protection problem, in which unprotected chromosome ends are detected as DNA double-strand breaks (DSBs) and erroneously repaired, yielding chromosome end-to-end fusions and driving the acquisition of genomic abnormalities that manifest many of the behaviors of cancer [21,22]. Shelterin assembly occurs hierarchically, with double-stranded telomere DNA first bound sequentially by telomeric repeat-binding factor 1 and 2 (TRF1 and TRF2) homodimers. Importantly, TRF1 aids in DNA replication through G-rich telomeric sequences [23]. Moreover, TRF1 and TRF2 binding protects against illicit DNA repair by shielding chromosome ends from DNA damage recognition [24] and facilitating telomere loop (t-loop) formation, whereby obligate 3′ single-stranded telomere overhangs are sequestered within triplex DNA structures [25]. These terminal single-stranded overhangs are further engaged by the ssDNA-binding protein protection of telomeres 1 (POT1), which coordinates chromosome nucleolytic end-processing following DNA replication and safeguards telomere ssDNA from DNA damage response (DDR) activation in conjunction with the shelterin component TPP1 [26] by opposing the activity of multiple DNA repair pathways [27,28,29]. Once bound to telomeric repeat sequences, TRF1 and TRF2 assemble the remaining shelterin components, which include POT1-TPP1, TRF1-interacting nuclear protein 2 (TIN2) and repressor/activator protein 1 (RAP1) [30,31]. In addition, shelterin binding serves to regulate recruitment of an array of accessory factors to telomeres. These include (i) alternative telomere dsDNA-binding proteins, such as TERB1/TERB2 [32] and TZAP, which preferentially binds to long telomeres and institutes an upper limit to telomere length via initiation of telomere trimming [33]; (ii) proteins involved in modulating DDRs, including the exonuclease Apollo, which processes telomere overhangs and prevents aberrant recombination-based DNA repair [34,35]; NBS1 and Ku70/Ku80, which collectively sense DSBs and coordinate end-resection and diminution of DSB repair at telomeres [24,36]; and the structure-specific endonuclease scaffold SLX4 together with its associated nucleases [37]; (iii) proteins that exert temporal control and impart strand specificity during telomere replication, namely the CTC1-STN1-TEN1 (CST) and DNA polymerase α-DNA primase (pol α-primase) complexes [38], as well as the DNA helicase RTEL1 [39]; and (iv) the poly(ADP-ribose) polymerase (PARP) homologs tankyrase 1 and tankyrase 2, which promote telomere maintenance by modifying TRF1 and liberating it from telomeres [40,41]. The telomere proteomic landscape is further defined by the cadre of histone proteins that imprint telomeric chromatin with specific architectures [42,43,44,45,46,47,48] or serve as components of telomere-processing enzyme complexes [49]. Collectively, shelterin and other telomere-binding proteins act as critical mediators to combat the end-replication and end-protection problems, thereby orchestrating the events that govern telomere homeostasis.

Telomeres buffer against the loss of genetic information during DNA replication and cell division by serving as a medium for genomic attrition. As a corollary, telomeres may counteract the end-replication problem by employing one or more telomere maintenance mechanisms (TMMs) that actively synthesize new telomere repeats. Telomeres are dynamically extended during embryonic development and in germ cell and stem cell populations postnatally but are otherwise not maintained in most cell types [50,51,52]. However, TMM reactivation occurs in cancer cells when their telomere length becomes critically short (longest telomere <12.8 repeats in length [53]; so-called telomere crisis), which induces genome instability and facilitates the transmission of DNA damage lesions that promote tumorigenesis [54,55,56]. Notably, telomere homeostatic factors act cooperatively with DDR proteins to bypass apoptosis secondary to telomere crisis, such that many cancers exhibit telomeres that are stably maintained close to their critical length [57,58]. In this way, both telomere shortening and extension serve as necessary steps in telomere-dependent malignant transformation. Currently, two disparate mechanisms for telomere elongation exist, namely telomerase and Alternative Lengthening of Telomeres (ALT). Telomerase is a ribonucleoprotein (RNP) made up of a core dimer consisting of a reverse transcriptase (RT) protein component (TERT) and an RNA component (TR or TERC) that serves as a template for RNA-dependent DNA synthesis [49]. In contrast, ALT relies on homologous recombination-mediated invasion of contiguous telomeres for templated DNA synthesis in a manner that is highly reminiscent of break-induced replication [59]. The vast majority of cancers appear to engage telomerase for telomere extension. Importantly, the molecular constituents of telomerase function as additional telomere accessory factors that affect the replicative and protective functions of telomeres, frequently doing so in conjunction with shelterin or other core telomere-binding proteins. In addition, telomerase possesses a host of extratelomeric (i.e., telomere-independent) functions that influence telomere homeostasis, as well as signal transduction, cellular energetics, and transcriptional regulation of gene expression [60]. In this review, we outline the pathways controlling the expression, assembly, and function telomerase, as well as discuss the telomeric and extratelomeric mechanisms connecting telomerase to the hallmarks of cancer. Finally, we examine how these mechanistic insights can facilitate the development of telomere-directed cancer therapies.

## 2. Regulation of Telomerase Component Expression and Function

### 2.1. Transcriptional Regulation of TERT

**Figure 1:**
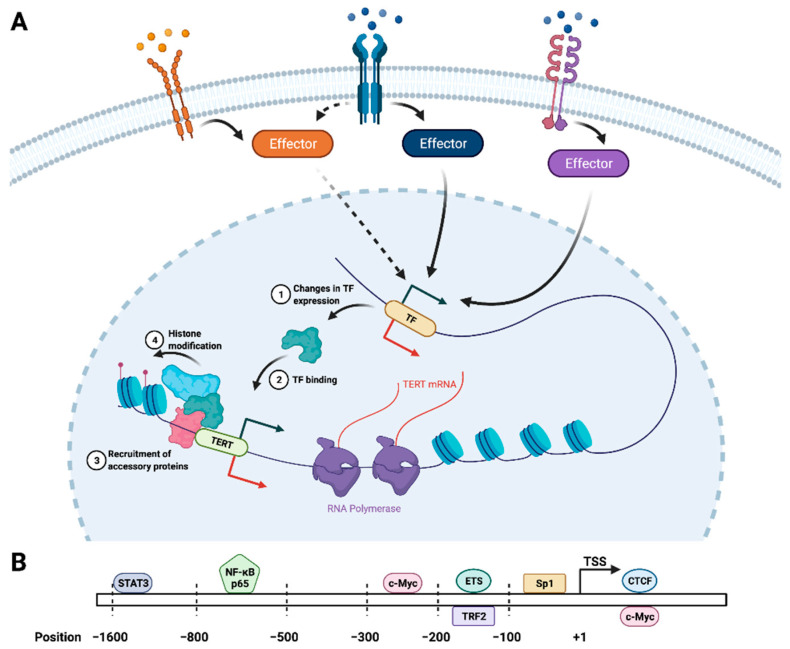
Transcriptional regulation of TERT. (**A**) TERT transcription is mediated by the coordinated actions of myriad intracellular signaling pathways that are initiated by specific ligands (e.g., growth factors, cytokines). The downstream effectors of these receptor-ligand interactions activate (green arrow) or repress (red arrow) the expression of TERT as well as TFs that regulate TERT expression (**1**). As a consequence of transcriptional up- or downregulation, these TFs (represented by the green protein) are more or less able to engage the *TERT* promoter, both on their own (**2**) and in conjunction with accessory transcriptional regulators (represented by the pink and blue proteins) (**3**). These accessory factors include histone-modifying enzymes, which alter the chromatin landscape at the *TERT* promoter to facilitate or inhibit transcription (**4**). The diversity of signaling inputs and TFs that oversee TERT transcription enable combinatorial control of telomerase function in response to intracellular and extracellular conditions. (**B**) The human *TERT* promoter harbors binding sites for the major transcriptional regulators of TERT expression [shown as approximate nucleotide position relative to the transcription start site (TSS)] [64,65,66,67,68]. TFs shown above and below the chromosome (rectangle) have binding motifs located on overlapping segment of the *TERT* promoter, rather than binding in opposing orientations.

In addition to the core TERT:TR dimer, telomerase is composed of (i) the dyskerin-NOP10-NHP2-GAR1 tetramer (also known as the H/ACA RNP complex) that functions in telomerase RNP biogenesis [61]; (ii) TCAB1, which recruits the telomerase core to subnuclear Cajal bodies (CBs) for further processing by the H/ACA complex [62]; and (iii) the histone proteins H2A and H2B, which stabilize a critical TERT-interacting domain in TR during holoenzyme assembly [49]. TERT and TR are necessary to carry out telomere extension by telomerase [63]; thus, the expression of these components, particularly TERT, is under tight transcriptional control downstream of a host of intracellular signaling pathways that mediate changes in chromatin structure and transcription factor (TF) binding (Figure 1).

While the identity of TERT transcriptional regulators can be highly cancer-specific [69], there exists a set of shared TFs that serve to influence TERT expression across numerous cancer types. The most well-characterized of these is the Myc/Max/Mad1 family of TFs, which bind to specific sequences in the *TERT* promoter and coordinate the recruitment of additional TFs and chromatin-modifying enzymes that modulate *TERT* promoter accessibility and gene expression [70,71]. Moreover, c-Myc sits at the convergence of multiple signaling pathways, which allows for fine-tuning of TERT expression and telomerase activity in response to specific cellular and environmental cues. For instance, NF-κB functions broadly during malignant transformation, tumor progression, and modulation of the tumor immune milieu; it also directly stimulates TERT expression [67,72] and upregulates c-Myc, thereby potentiating the transcriptional effects of c-Myc on TERT [66]. Along these lines, members of the signal transducer and activator of transcription (STAT) family of TFs, which function downstream of a variety of cytokine and growth factor receptors, activate both TERT and c-Myc transcription in cancer cells [65,73,74]; they also bind cooperatively with c-Myc to regulate target gene expression [75]. Similar cooperativity occurs between c-Myc and specificity protein (Sp) family TFs, particularly Sp1 [76], which exhibits differential expression in numerous tumor types and is functionally implicated in driving cancer phenotypes [77]. Moreover, c-Myc and Sp1 mediate the transcriptional responses brought about by engagement of mitogenic and pro-survival signaling pathways, including epidermal growth factor (EGF) [78], vascular endothelial growth factor (VEGF) [79,80], transforming growth factor β (TGF-β) [81], Wnt/β-catenin [82,83,84], and NF-κB. Importantly, numerous cancer types harbor monoallelic *TERT* promoter mutations that create de novo TF binding sites, namely for members of the ETS family of TFs (reviewed in [85,86]). Engagement of the *TERT* promoter by ETS proteins stimulates *TERT* transcription, providing a mechanistic connection between cancer associated *TERT* mutations and telomerase reactivation. Of note, aberrant overexpression from the mutant *TERT* promoter is further enhanced by the upregulation of several ETS TFs through mitogen-activated protein kinase (MAPK) signaling [87,88]. Conversely, effectors that diminish TERT expression, which include several important tumor suppressors, become dysregulated in cancer cells as a means of de-repressing telomerase activity. For example, exchanging c-Myc for Mad1 at the *TERT* promoter results in decreased TERT expression via direct competition between the two proteins for at the *TERT* promoter binding [71,89]. This competition has a dual effect by simultaneously reversing the transcriptional activation effects of c-Myc, while also actively inhibiting gene expression through Mad1-mediated recruitment of transcriptional repressors. TERT expression is similarly reduced by the DNA damage-responsive cell cycle regulator p53, one of the most commonly mutated tumor suppressors across diverse cancer types. Specifically, p53 interacts with Sp1 at the TERT promoter, an interaction that is sufficient to abrogate TERT expression [90,91]. Another tumor suppressor, CCCTC-binding factor (CTCF), blocks TERT expression by binding within its open reading frame and physically displacing the transcription machinery [92,93]. Intriguingly, the telomere-binding protein TRF2 exerts direct transcriptional control over TERT expression by binding specific DNA secondary structural elements and recruiting the polycomb repressive (PRC) complex to the *TERT* promoter [64], suggesting that telomere-binding proteins act as critical regulators of telomerase activity through both telomere-dependent and -independent mechanisms. Taken together, these findings illuminate the myriad pathways that integrate intracellular and extracellular signals to enable combinatorial control of telomerase function.

Many of the events that characterize the TERT transcriptional landscape are mediated by epigenetic modifiers that are differentially recruited or activated by specific TFs. These include enzymes that modify both DNA and histones with an assortment of chemical moieties (Figure 1). For instance, the relative binding of c-Myc versus Max1 at the *TERT* promoter modulates the recruitment of histone acetyltransferase (HAT) and histone deacetylase (HDAC) co-regulatory proteins, which in turn influences TERT expression [71]. More broadly, numerous TFs recruit HAT and HDAC proteins to the *TERT* locus [94]. Similarly, specific histone methytransferases share overlapping binding sites in the *TERT* promoter with CTCF. Once bound, these enzymes catalyze targeted lysine trimethylation on histone H3 (H3K4me3), which facilitates TERT expression by eliciting optimal TF occupancy at the *TERT* promoter [95]. Histone methylation dynamics play a parallel role in suppressing TERT expression through the actions of the non-metastatic 2 (NME2; also known as nucleoside diphosphate kinase 2) metastasis suppressor protein [96]. Specifically, NME2 recruits the RE1-silencing transcription factor (REST)–lysine-specific histone demethylase 1 (LSD1) co-repressor complex to the *TERT* promoter, where it erases activating methyl marks on histones to transcriptionally silence TERT [97]. Notably, this transcriptional function of NME2 is augmented by its direct association with the telomerase holoenzyme and inhibition of telomerase activity [98]. Relatedly, the *TERT* promoter undergoes extensive DNA methylation, which quite paradoxically stimulates gene expression. The mechanism for this is incompletely understood but may involve blocking CTCF recruitment secondary to methylation of CG-rich DNA sequences in the *TERT* promoter [92,99]. Collectively, the covalent modification of DNA and histones exerts powerful control over the set of TFs bound at the *TERT* genomic locus, which in turn dictates TERT expression and telomerase function. Intriguingly, given the presence of histone proteins in the telomerase holoenzyme structure [49], histone modification may play an as-yet-unexplored role in telomerase regulation at the post-translational level as well.

### 2.2. Post-Transcriptional Regulation of TERT and TR

**Figure 2:**
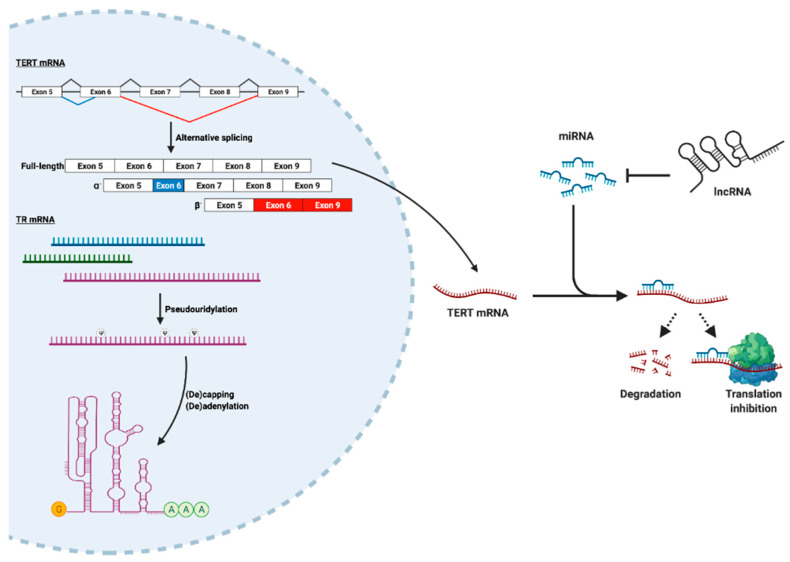
Post-transcriptional regulation of TERT and TR. Both TERT and TR undergo substantial post-transcriptional processing that is required for telomerase maturation and activity. TERT is expressed as multiple alternatively spliced isoforms, most notably the catalytically inactive α^-^ and β^-^ variants. Shown are exons 5–9 of the *TERT* primary transcript, with canonical splice junctions shown in black and alternative splice junctions corresponding to α^-^ and β^-^ shown in blue and red, respectively, along with their corresponding mature mRNAs. *TERT* mRNA is also targeted by multiple miRNAs, which hybridize to complementary sequences in the *TERT* transcript and suppress TERT expression via RNA degradation or translational repression. Paradoxically, some TERT-targeting miRNAs can also stimulate TERT expression (see text). In turn, miRNA availability is regulated by other noncoding RNAs, including lncRNAs. In contrast, TR is synthesized as a pool of alternative transcripts (represented in blue, green, and purple). TR subsequently undergoes extensive pseudouridylation (Ψ), which may be important for telomerase assembly, activity, or processivity. In addition, TR undergoes addition and removal of a 5′ cap (represented by the yellow “G”) and 3′ oligo-adenosine tail (represented by the green “A” sequence), whose presence or absence dictate TR stability and dramatically influence telomerase function.

In addition to transcription, TERT abundance is heavily influenced by post-transcriptional regulatory mechanisms. Importantly, these mechanisms also play a central role in TR processing and function (Figure 2). First, *TERT* mRNA is subject to extensive alternative splicing, with the functions of many of these splice variants remaining to be elucidated [100]. Two variants that have been extensively studied, termed α- and β-, both disrupt TERT translation and preclude telomerase activity [101,102]. While the α-variant is missing a critical motif in its RT domain that imparts a dominant-negative effect on telomerase [103], the β- variant reduces telomerase activity via nonsense-mediated decay while simultaneously shielding cancer cells from treatment-induced apoptosis [102]. Interestingly, other TERT variants that lack catalytic activity can still stimulate cell proliferation [9]. In this way, alternative splicing of TERT can mobilize multiple mechanisms that endow cells with malignant phenotypes. Moreover, TERT alternative splicing is coordinated by many of the key signaling effectors that control TERT transcription, including c-Myc and TGF-β [104]. In addition to alternative splicing, TERT expression is post-transcriptionally regulated through the actions of noncoding RNAs (ncRNAs), namely microRNAs (miRNAs). Typically, cognate miRNAs bind the TERT 3′-UTR to suppress its expression via RNA degradation or repression of translation. However, a subset of miRNAs can paradoxically upregulate TERT [105]. Likewise, TERT expression can be indirectly influenced by miRNAs through alterations in the abundance of TERT transcriptional regulators [106]. miRNAs, in turn, interact with other ncRNAs to modulate their availability [107,108]. Additional regulation of telomerase by ncRNAs is carried out by the telomeric repeat containing RNA (TERRA), which is the product of telomere DNA transcription [109]. Specifically, TERRA binds TERT and base-pairs with TR to inhibit telomerase activity [110]. In addition, TERRA has been shown to form DNA-RNA hybrid structures, known as R-loops, at telomeres [111]. These structures may play a dual role in blocking telomerase by (i) recruiting histone-modifying enzymes that induce telomeric heterochromatin [112], which can attenuate extension by telomerase [113]; and (ii) promoting telomere extension by homologous recombination (i.e., ALT) [114]. Collectively, the combined actions of RNA splicing and ncRNAs exert critical control over TERT abundance and telomerase function.

The mechanisms that dictate TERT expression are reminiscent of those that regulate TR, with TR undergoing additional post-transcriptional processing as well (Figure 2) [115]. For instance, TR is expressed as a heterogeneous pool of transcript isoforms that are post-transcriptionally processed to a mature form [116,117]. The stability and utility of alternative and pre-processed TR transcripts remains an active area of investigation. In contrast to TERT mRNA, TR requires extensive chemical modification in order to optimize its function. At least six nucleotides in TR undergo pseudouridylation, including two sites that lie within a highly conserved domain essential for telomerase catalytic activity. Although in vitro reconstitution with synthetically pseudouridylated TR results in modest alterations in telomerase activity and processivity, the relevance of these modifications to telomerase function in vivo remains an open question [118]. Furthermore, TR precursors undergo cycles of oligo-adenylation and de-adenylation, which together control the steady-state levels of TR. Oligo-adenylation is carried out primarily by the poly(A) polymerase PAPD5 [119], while de-adenylation is performed on oligo-adenylated intermediates by poly(A) ribonuclease (PARN) [120,121]. In the absence of de-adenylation, TR intermediates initially accumulate and are subsequently degraded within RNA exosomes [122], resulting in reduced telomerase activity. In parallel, TR acquires a 5′ cap that is hypermethylated (i.e., m^2,2,7^G) by the enzyme trimethylguanosine synthase (TGS1) [123]. Hypermethylation leads to TR decay, while TGS1 deficiency increases telomere length and telomerase activity [124], indicating that TR capping and adenylation negatively regulate telomerase. Taken together, telomerase biogenesis and activity are profoundly impacted by post-transcriptional regulation of its core components through both shared and distinct pathways.

### 2.3. Regulation of Other Telomerase Components

In comparison to TERT and TR, far less is known about the regulation of other telomerase components. Moreover, it is important to note that many of these accessory components also have diverse cellular functions unrelated to telomere homeostasis. For example, two members of the H/ACA RNP complex, dyskerin and GAR1, are post-translationally modified by PARP1, an event that may promote telomerase assembly and activity [125,126]. However, the H/ACA complex is also responsible for catalyzing pseudouridylation, the most abundant post-transcriptional RNA modification in cells [127]. By exerting quality control over the stability and function of a variety of cellular RNAs, including tRNAs, rRNAs, and the RNA constituents of the spliceosome, the H/ACA complex possesses broad functionality in promoting fidelitous gene expression. For instance, TCAB1 recruits telomerase to CBs, which are sites of assembly for spliceosomal snRNPs [128,129]. Furthermore, CBs appear to nucleate chromosomal clusters that contain genomic loci rich in histone genes, thereby influencing their expression [130]. Coupled with the presence of histone proteins in the telomerase holoenzyme, these findings emphasize the critical functional diversity of all of the telomerase accessory components. Thus, it is conceivable that expression of these factors is less stringently regulated than TERT and TR, or alternatively, that the mechanisms governing the expression of these factors is disjoint from those overseeing TERT and TR. Future studies need to examine whether, and by what means, accessory proteins function as regulatory nodes for telomerase biogenesis in a manner akin to TERT and TR.

## 3. Extratelomeric Functions of Telomerase: Implications for Cancer Initiation and Progression

The ability of telomerase to execute its role in telomere homeostasis is dependent not only on the proper expression and assembly of its constituents, but also on its recruitment to and engagement of the telomere template for DNA synthesis. Accordingly, telomerase recruitment and processivity are dictated by the presence of specific telomere-binding proteins—namely, the shelterin components POT1 [131,132], TPP1 [133], and TIN2 [134], and by the resolution of higher-order telomere DNA structures through differential protein binding [135,136] or post-translational modification [137]. The multifaceted nature of telomerase recruitment and catalytic activity can be viewed as a series of bifurcation points, at which the constituents of telomerase can either (i) assemble sequentially into active holoenzyme units, (ii) adopt and execute alternative functions via recruitment to other genomic loci, or (iii) form complexes with discrete binding partners throughout the cell. As outlined in the succeeding sections, these “extratelomeric” functions of telomerase drastically expand its functional repertoire to encompass regulation of signal transduction, metabolism, and gene expression.

### 3.1. TERT as a Transcription Factor and Signaling Effector

**Figure 3:**
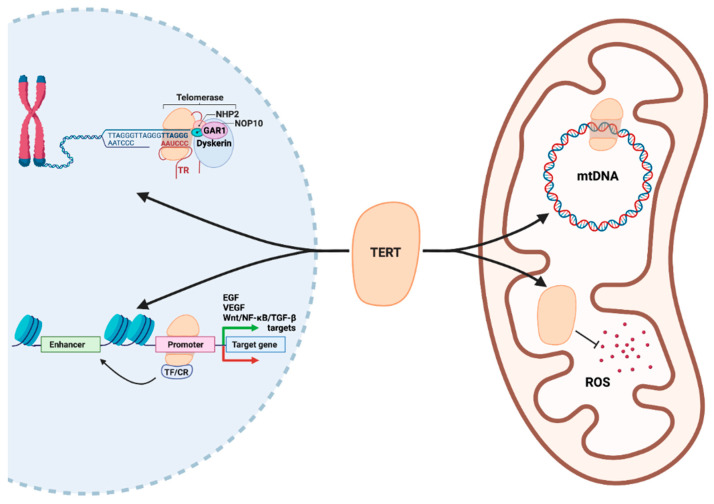
Extratelomeric functions of TERT. In the nucleus (**left**), TERT carries out its canonical role in telomere extension in complex with the other components of the telomerase holoenzyme (TR, dyskerin, GAR1, NOP10, NHP2). In addition, TERT binds to the promoters of genes that encode key growth factors (EGF, VEGF), as well as secondary effectors of tumor-promoting signaling pathways (Wnt, NF-κB, TGF-β), thereby positively or negatively regulating their expression. These transcriptional effects are frequently mediated by the actions of TFs or chromatin remodelers (CR) that complex with TERT at its target loci and influence both local and distant chromatin structure (i.e., at enhancers). In mitochondria (**right**), TERT possesses transcriptional, metabolic, and detoxification functions. In particular, TERT recognizes specific sites in the mitochondrial genome (mtDNA), which both alters mitochondrial gene expression and shields mtDNA from damage. Additionally, TERT plays a DNA-independent role in the neutralization of reactive oxygen species (ROS), which otherwise induces DNA damage and institute a host of aberrations in cellular metabolism and survival.

In addition to endowing transformed cells with replicative immortality, telomerase also regulates multiple processes of tumor formation and progression by acting as a TF (Figure 3). Indeed, studies examining the relationship between telomerase activity and transcriptomic alterations in cancer cells have revealed a host of genes whose transcription appears to be directly overseen by TERT, including EGFR and VEGF [12,138]. In line with this, TERT binds to the VEGF promoter [139] and functions as a downstream effector of VEGF signaling [140], while inhibition of telomerase abrogates VEGF expression and angiogenesis [141,142]. Additionally, TERT exhibits functionally significant intersections with the Wnt/β-catenin pathway. Specifically, TERT complexes with the chromatin remodeler SMARCA4 to induce the expression of Wnt-responsive genes that promote tumor formation, such as c-Myc and VEGF [143,144,145]. Conversely, TERT expression is reciprocally regulated by β-catenin [146], which may sustain a positive feedback loop between Wnt signaling and TERT transcriptional activity. Similarly, TERT can bind NF-κB following nuclear import and modulate the expression of specific NF-κB-dependent target genes that orchestrate both cell-intrinsic and -extrinsic mechanisms of tumor progression [14,147]. Moreover, NF-κB acts in conjunction with TGF-β to drive cancer cell dissemination and disease progression, which is potentiated by TERT through its interaction with the TGF-β-responsive TF, ZEB1 [148,149]. The signaling inputs that modulate the transcriptional versus canonical functions of TERT are presently unknown. However, given that TERT frequently operates within transcriptional feedback loops, it is conceivable that signals that activate these loops simultaneously promote the extratelomeric functions of TERT. For example, cytokines such as interleukin 6 (IL-6), insulin-like growth factor 1 (IGF-1) [150], and tumor necrosis factor α (TNF-α) [14] stimulate both NF-κB transcriptional activity and telomerase function. Similarly, Wnt ligands released within the tumor microenvironment [151] may drive TERT expression without compensatory upregulation of other telomerase components [146]. Taken together, these findings underscore the ability of TERT to function within a transcriptional nexus controlled by numerous oncogenic signaling inputs.

### 3.2. TERT as a Regulator of Cellular Energetics

Tumor formation and progression are associated with dramatic shifts in nutrient utilization and energy mobilization as cells traverse the path from healthy tissue to aggressive disease [152]. This metabolic derangement of cell bioenergetics is mediated by altered mitochondrial function, which impacts cancer cell redox homeostasis, intracellular signaling, and survival [153]. Importantly, mitochondrial function is reliant upon the expression and subcellular localization of TERT, which shields the mitochondrial genome from damage and buffers cells against oxidative stress (Figure 3; [154,155]). At a molecular level, TERT acts synergistically with the master regulator of mitochondrial biogenesis, PCG-1α, thereby exerting direct transcriptional control over mitochondrial function [156]. In addition, TERT plays a role in coordinating the expression of the mitochondrial genome, which serves as a critical determinant of cellular metabolism [157]. Furthermore, TERT is capable of binding to mitochondrial DNA (mtDNA) and maintaining genome integrity under oxidative stress, in part by shielding mtDNA from oxidative damage [154] as well as alleviating reactive oxygen species (ROS) production through upregulation of superoxide dismutase [158] and components of the electron transport chain [159]. Thus, protection from oxidative stress appears to occur through both metabolic and transcriptional mechanisms that are initiated by TERT. Notably, exposure to ROS generated within primary versus metastatic sites can have differential effects on cancer cell growth and metastatic dissemination [160,161]. More broadly, telomerase activation in cancer cells alters the catabolic-anabolic balance for both lipids [162] and carbohydrates [156,163,164], which may in turn regulate mitochondrial biogenesis [165]. Furthermore, TERT mediates nuclear-mitochondrial crosstalk that is critical for circumventing potentially deleterious DNA damage that would otherwise induce apoptosis [7]. Taken together, these findings highlight the ability to TERT to oversee multiple metabolic and cellular detoxification functions that substantially influence cancer cell survival, adaptation, and dissemination.

### 3.3. Telomerase, DNA Damage, and Genome Stability

Through their role in chromosomal end-protection, telomeres defend against genomic instability by masking chromosome ends from constitutive exposure to the DDR machinery [22,166]. Moreover, in spite of the protection afforded by the suite of shelterin and other telomere-binding proteins, telomeres possess features of chromosomal fragile sites and are susceptible to replication stress [23,56]. During malignant transformation, telomerase activation frequently occurs in the context of telomere crisis, which facilitates the stable propagation of cancer-promoting genomic rearrangements while limiting the excessive accumulation of senescence-inducing mutations and structural aberrations [167]. Thus, telomerase plays a central part in maintaining the delicate balance between adaptive genome instability and maladaptive replication stress in the context of tumor initiation and progression. Along these lines, telomerase activation is sufficient to reduce replication stress brought on by oncogene induction [168], aneuploidy, or the administration of DNA-damaging agents [169]. At a molecular level, TERT can bind and repair collapsed replication forks at stressed telomeres by coordinating recruitment of the DNA repair machinery [170], although this mechanism has not yet been observed in humans. Conversely, the absence of TERT impairs the ability of cells to recognize and repair DNA damage lesions, including lesions that are extratelomeric [6]. Intriguingly, this DNA repair deficiency is independent of alterations in telomere length. Collectively, these results suggest parallel telomere-dependent and -independent roles for TERT in orchestrating cellular responses to DNA damage and consequent replication stress.

### 3.4. Extratelomeric Functions of TR and Other Telomerase Components

As discussed above, the non-TERT protein components of telomerase perform a wide array of telomere-independent functions, such as RNA editing and the assembly of RNA-protein complexes. In contrast, the existence and nature of extratelomeric functions for TR are not well-understood but are beginning to be uncovered. Indeed, upregulation of TR during tumorigenesis can occur in the absence of increased telomerase activity [171], suggesting that TR possesses telomerase-independent functions in cancer cells (Figure 3). In support of this, genome-wide mapping of ncRNA occupancy identified a TR-binding motif that was present at loci encoding Wnt and c-Myc signaling effectors, and at NF-κB targets [172,173]. Moreover, TR can also be trafficked into mitochondria, where it is nucleolytically processed and re-exported into the cytosol [174]. The function of this processed form of TR remains unclear, although its abundance is responsive to mitochondrial oxidative activity. An alternative mechanism underlying the extratelomeric functions of TR is based on the existence of an optional open reading frame within the TR primary transcript, whose translation produces a 13 kDa protein called hTERP (human TElomerase RNA Protein) that protects cells against apoptosis [175]. Along these lines, TR mutants deficient in TERT binding still protect cells against drug-induced apoptosis via hTERP production. Moreover, TR knockdown promotes drug-induced apoptosis in the absence of detectable telomere shortening, while TERT knockdown has no impact on cell survival, findings consistent with the anti-apoptotic actions of TR being dependent on its translation [176]. Thus, TR harbors many of the same extratelomeric functions as TERT, but the mechanisms underlying these functions may be distinct and complementary. As a consequence, both core components of telomerase may independently and synergistically orchestrate the acquisition of aggressive cancer traits, particularly dysregulated cell proliferation and survival.

## 4. Clinical Applications of Telomeres and Telomerase in Oncology

In spite of the mechanistic insights gained through molecular interrogations of telomerase, much work remains to translate these insights into clinical action. Nevertheless, the determination of telomere homeostatic mechanisms in individual tumors presents a promising avenue for improved cancer diagnosis, prognosis, and therapy. Indeed, shorter telomere length is observed in tumor cells compared to adjacent normal tissue in biopsy specimens [177]. However, the relationship between telomere length and disease progression is not as clear, as some cancers exhibit a positive correlation between telomere length and disease stage [177], while others reflect a negative correlation [178,179]. These inconsistencies may stem from differences in telomere dynamics across distinct tissues, or from unique interactions between telomere and cellular homeostatic mechanisms that are characteristic of particular tumor types. Moreover, telomere lengths in tumors and surrogate tissues may be employed to predict patient survival [180], as well as to assess patient response to therapy [181]. In addition to telomere length, TMM identity can be directly assayed in individual tumors and associated with pathologic and clinical features, including survival and metastasis [182,183]. Similar approaches can be employed to quantify TERRA expression, whose decreased expression is associated with poor survival and increased progression to metastasis across multiple cancer types [184,185,186]. Relatedly, the presence of mutations, rearrangements, or duplications that hyperactivate telomerase are pathognomonic in numerous cancers, where they upregulate *TERT* through a variety of genetic and epigenetic mechanisms [187,188]. Importantly, the presence of specific TMMs and their underlying genomic aberrations may presage potential therapeutic vulnerabilities. Future studies designed to systematically catalog the connections between telomere length, TMM identity, and disease progression across cancers are necessary to shed additional light on the nature of telomere homeostasis in specific tumor types, and to provide a foundation for personalized diagnosis and prognosis based on telomere features.

Recent efforts aimed at the therapeutic targeting of telomerase have been centered on enzyme inhibition, cytotoxic substrate incorporation, telomere destabilization, and anti-telomerase immunotherapy [189]. Enzyme inhibitors include both small molecules and TR template antagonists, both of which have demonstrated antitumor activity in multiple preclinical cancer models [190,191,192]. Moreover, the inhibitors BIBR1532 and GRN163L (also known as Imetelstat) have been assessed in several clinical trials across diverse cancer types and in recurrent and metastatic disease settings [193]. To date, these trials have revealed modest benefit over standard-of-care, although a handful of trials have demonstrated a potential telomere length-dependent therapeutic effect [194], an effect that is likely attributable to the inherently complex relationship between telomere length and tumor progression. In addition, it is possible that telomerase-driven tumors adaptively activate secondary TMMs, such as ALT, in response to treatment, as previously observed in the context of experimental abrogation of telomerase [195,196,197]. Such a phenomenon could explain the heretofore observed lack of therapeutic benefit from telomerase inhibitors and would provide a basis for examining the efficacy of combinatorial therapies targeting multiple TMMs [198]. As noted above, telomere crisis is a necessary step not only in TMM reactivation and tumorigenesis, but also in the acquisition of metastatic phenotypes [198,199]. As such, the administration of telomerase inhibitors must be precisely timed to avoid facilitating telomere length-dependent disease progression, which manifests as treatment resistance. To this end, small molecule inhibitors of other telomeric proteins, such as tankyrase 1, may reduce resistance when administered in combination with telomerase inhibitors, thereby enhancing telomere attrition and cell death [200]. Notably, these effects appear to be driven not only by diminution of telomerase activity, but also by a reduction in Wnt signaling that provides for a complementary, length-independent mechanism for anti-telomerase therapy [201,202].

An orthogonal approach to telomerase inhibition involves the administration of small molecules that act as high-affinity substrates for telomerase, but instead activate cytotoxic DDRs when they are incorporated into telomere DNA [203]. Such agents exhibit high selectivity for cells that harbor active telomerase, thus limiting their off-target effects. Importantly, this approach has a distinct advantage over traditional pharmacologic inhibition of telomerase in that therapy-induced cell death occurs much faster, thereby minimizing the risk of developing resistance. Accordingly, cytotoxic telomerase substrates have shown marked efficacy in preclinical models of breast [198], lung, colon, and pancreatic cancers [203]. Moving forward, more research is needed to better determine the in vivo pharmacokinetic and pharmacodynamic properties of these agents, as well as their safety and efficacy in patients. In a similar vein, telomere destabilization can be accomplished by introducing mutant-template TR, which causes misincorporation during telomere DNA synthesis that results in cell death or increased sensitivity to other anti-cancer agents [204,205]. The effects of telomere destabilization have been investigated preclinically; however, clinical implementation of such modalities may be clinically intractable and will require additional research on the optimal delivery, expression, and incorporation of mutant TR templates in vivo.

Telomerase can be viewed as a tumor-associated neoantigen that, when leveraged therapeutically, may elicit potent anti-tumor immune responses. Current investigational telomerase immunotherapeutic platforms include peptide and dendritic cell (DC) vaccines, both of which are being assessed in diverse preclinical and clinical settings [206]. Peptide vaccines consist of short amino acid chains derived from the full-length TERT sequence that are administered as single agents or in combination with immune modulatory or other anti-cancer agents. TERT peptide vaccines, including GV1001 [207], GX301 [208], and Vx-001 [209], are displayed on the surface of antigen-presenting cells, which express major histocompatibility complex class II (MHCII) proteins that are responsible for coordinating robust, antigen-specific cell-mediated and humoral immune responses through the activation of CD4^+^ T cells and B cells, respectively. Importantly, multiple clinical trials have revealed that these vaccines are generally safe and well-tolerated; they also yield survival benefits for patients across a host of tumor types that correlate with the strength of antitumor immune responses [206]. On the other hand, DC vaccines introduce a concentrated population of dendritic cells that have been engineered to express, process, and present specific tumor-associated antigens. Through the activation of naïve T cells, DCs initiate and orchestrate an adaptive immune response against cancer cells [210]. In preclinical [211] and clinical studies [212], TERT DC vaccines stimulated cytotoxic T cell responses and induced immune memory. Indeed, in clinical trials involving multiple cancer types, patients achieved an immunologic response [206], with further analyses revealing putative disease stabilization and increased disease-free survival [213,214,215]. Of note, peptide and DC vaccines have been assessed specifically in the context of metastatic disease, where they have shown promise despite lingering questions related to their general efficacy in metastatic settings [216,217]. More recently, the effectiveness of telomerase DNA vaccines has been examined [218,219], as has the feasibility of adoptive transfer of anti-telomerase chimeric antigen receptor (CAR) T cells [220]. It should be noted that, while telomerase is not widely expressed throughout the body, its activity is maintained by tissue-resident stem cell populations, including hematopoietic [221], intestinal [222], and germline [223]. It remains to be seen whether therapies designed to eradicate telomerase-positive cancer cells impact the survival and regenerative capacity of these stem cell populations. However, despite the difficulties inherent in these approaches, telomerase remains an appealing therapeutic target in need of continued innovation.

Targeting the extratelomeric functions of telomerase poses an exciting alternative approach to telomere-directed therapy, but one that is accompanied by formidable challenges. First and foremost, our knowledge of the molecular mechanisms underlying these pathways remains incomplete, which limits our ability to rationally design targeted therapies. Critically, however, cancer cells that are exposed to traditional telomerase-targeting therapies may activate resistance mechanisms that enable not only their continued survival, but also their acquisition of highly aggressive features [198,224,225]. These findings suggest that specific targeting TMM plasticity may best be achieved using a multimodal strategy. As a corollary, combinatorial telomeric and extratelomeric approaches may serve to effectively eradicate telomerase-driven cancers, while limiting their ability to give rise to resistant and recurrent disease [226,227]. Moving forward, significant attention should be devoted to (i) developing improved methods for measuring and understanding patient telomere length and TMM status, both at the time of diagnosis and longitudinally; (ii) exploring improved therapies that target the canonical and extratelomeric functions of telomerase; and (iii) propelling these therapies into clinical trials.

## 5. Conclusions

Telomeres are exquisitely complex structures whose composition and length are dynamically organized by a host of factors, notably the core and accessory components of telomerase. The abundance of these components and their ability to be assembled into telomerase holoenzyme units significantly impacts telomere homeostasis and the acquisition of cancer phenotypes. These effects are mediated not only by the enzymatic activity of telomerase, but also by the heterogeneous extratelomeric functions that are carried out by individual telomerase components. Regulation of telomerase expression, assembly, and function occur at all levels of gene expression and is governed by a range of intracellular and environmental stimuli that share substantial overlap with pathways that control malignant transformation and tumor progression. This regulatory diversity has major implications for telomere maintenance and underscores the immense potential for incorporating telomeres into clinical oncology paradigms through precision diagnostics and the design of novel therapies that target both telomere-dependent and -independent functions of telomerase.

## Abbreviations

ALT	Alternative Lengthening of Telomeres
CAR	chimeric antigen receptor
CB	Cajal body
CST	CTC1-STN1-TEN1
CTCF	CCCTC-binding factor
DC	dendritic cell
DDR	DNA damage response
DSBs	DNA double-strand breaks
EGF	epidermal growth factor
HAT	histone acetyltransferase
HDAC	histone deacetylase
hTERP	human telomerase RNA protein
miRNA	microRNA
ncRNA	noncoding RNA
PARN	poly(A) ribonuclease
PARP	poly(ADP-ribose) polymerase
POT1	protection of telomeres 1
RAP1	repressor/activator protein 1
RNP	ribonucleoprotein
RT	reverse transcriptase
Sp	specificity protein
TERRA	telomeric repeat containing RNA
TERT	protein component of telomerase
TF	transcription factor
TGF-β	transforming growth factor-β
TIN2	TRF1-interacting nuclear protein 2
TMM	telomere maintenance mechanism
TR or TERC	RNA component of telomerase
TRF1	telomeric repeat-binding factor 1
TRF2	telomeric repeat-binding factor 1
VEGF	vascular endothelial growth factor

## References

[1] Herbert B., Pitts A.E., Baker S.I., Hamilton S.E., Wright W.E., Shay J.W., Corey D.R. (1999). Inhibition of human telomerase in immortal human cells leads to progressive telomere shortening and cell death. Proc. Natl. Acad. Sci. USA.

[2] Hanahan D., Weinberg R.A. (2011). Hallmarks of cancer: The next generation. Cell.

[3] Harley C.B., Futcher A.B., Greider C.W. (1990). Telomeres shorten during ageing of human fibroblasts. Nature.

[4] Hastie N.D., Dempster M., Dunlop M.G., Thompson A.M., Green D.K., Allshire R.C. (1990). Telomere reduction in human colorectal carcinoma and with ageing. Nature.

[5] Kelland L. (2007). Targeting the limitless replicative potential of cancer: The telomerase/telomere pathway. Clin. Cancer Res. Off. J. Am. Assoc. Cancer Res..

[6] Masutomi K., Possemato R., Wong J.M., Currier J.L., Tothova Z., Manola J.B., Ganesan S., Lansdorp P.M., Collins K., Hahn W.C. (2005). The telomerase reverse transcriptase regulates chromatin state and DNA damage responses. Proc. Natl. Acad. Sci. USA.

[7] Singhapol C., Pal D., Czapiewski R., Porika M., Nelson G., Saretzki G.C. (2013). Mitochondrial telomerase protects cancer cells from nuclear DNA damage and apoptosis. PLoS ONE.

[8] Choi J., Southworth L.K., Sarin K.Y., Venteicher A.S., Ma W., Chang W., Cheung P., Jun S., Artandi M.K., Shah N. (2008). TERT promotes epithelial proliferation through transcriptional control of a Myc- and Wnt-related developmental program. PLoS Genet..

[9] Hrdlickova R., Nehyba J., Bose H.R. (2012). Alternatively spliced telomerase reverse transcriptase variants lacking telomerase activity stimulate cell proliferation. Mol. Cell. Biol..

[10] Stampfer M.R., Garbe J., Levine G., Lichtsteiner S., Vasserot A.P., Yaswen P. (2001). Expression of the telomerase catalytic subunit, hTERT, induces resistance to transforming growth factor beta growth inhibition in p16INK4A(-) human mammary epithelial cells. Proc. Natl. Acad. Sci. USA.

[11] Cao Y., Li H., Deb S., Liu J.P. (2002). TERT regulates cell survival independent of telomerase enzymatic activity. Oncogene.

[12] Zhou L., Zheng D., Wang M., Cong Y.S. (2009). Telomerase reverse transcriptase activates the expression of vascular endothelial growth factor independent of telomerase activity. Biochem. Biophys. Res. Commun..

[13] George J., Banik N.L., Ray S.K. (2009). Combination of hTERT knockdown and IFN-gamma treatment inhibited angiogenesis and tumor progression in glioblastoma. Clin. Cancer Res. Off. J. Am. Assoc. Cancer Res..

[14] Ghosh A., Saginc G., Leow S.C., Khattar E., Shin E.M., Yan T.D., Wong M., Zhang Z., Li G., Sung W.K. (2012). Telomerase directly regulates NF-kappaB-dependent transcription. Nat. Cell Biol..

[15] Liu Z., Li Q., Li K., Chen L., Li W., Hou M., Liu T., Yang J., Lindvall C., Bjorkholm M. (2013). Telomerase reverse transcriptase promotes epithelial-mesenchymal transition and stem cell-like traits in cancer cells. Oncogene.

[16] Canela A., Vera E., Klatt P., Blasco M.A. (2007). High-throughput telomere length quantification by FISH and its application to human population studies. Proc. Natl. Acad. Sci. USA.

[17] Wright W.E., Tesmer V.M., Huffman K.E., Levene S.D., Shay J.W. (1997). Normal human chromosomes have long G-rich telomeric overhangs at one end. Genes Dev..

[18] Palm W., de Lange T. (2008). How shelterin protects mammalian telomeres. Annu. Rev. Genet..

[19] Watson J.D. (1972). Origin of concatemeric T7 DNA. Nat. New Biol..

[20] Soudet J., Jolivet P., Teixeira M.T. (2014). Elucidation of the DNA end-replication problem in Saccharomyces cerevisiae. Mol. Cell.

[21] McClintock B. (1941). The Stability of Broken Ends of Chromosomes in Zea Mays. Genetics.

[22] de Lange T. (2009). How telomeres solve the end-protection problem. Science.

[23] Sfeir A., Kosiyatrakul S.T., Hockemeyer D., MacRae S.L., Karlseder J., Schildkraut C.L., de Lange T. (2009). Mammalian telomeres resemble fragile sites and require TRF1 for efficient replication. Cell.

[24] de Lange T. (2018). Shelterin-Mediated Telomere Protection. Annu. Rev. Genet..

[25] Doksani Y., Wu J.Y., de Lange T., Zhuang X. (2013). Super-resolution fluorescence imaging of telomeres reveals TRF2-dependent T-loop formation. Cell.

[26] Rice C., Shastrula P.K., Kossenkov A.V., Hills R., Baird D.M., Showe L.C., Doukov T., Janicki S., Skordalakes E. (2017). Structural and functional analysis of the human POT1-TPP1 telomeric complex. Nat. Commun..

[27] Yang Q., Zheng Y.L., Harris C.C. (2005). POT1 and TRF2 cooperate to maintain telomeric integrity. Mol. Cell. Biol..

[28] Denchi E.L., de Lange T. (2007). Protection of telomeres through independent control of ATM and ATR by TRF2 and POT1. Nature.

[29] Palm W., Hockemeyer D., Kibe T., de Lange T. (2009). Functional dissection of human and mouse POT1 proteins. Mol. Cell. Biol..

[30] Ye J.Z., Donigian J.R., van Overbeek M., Loayza D., Luo Y., Krutchinsky A.N., Chait B.T., de Lange T. (2004). TIN2 binds TRF1 and TRF2 simultaneously and stabilizes the TRF2 complex on telomeres. J. Biol. Chem..

[31] Chen Y., Yang Y., van Overbeek M., Donigian J.R., Baciu P., de Lange T., Lei M. (2008). A shared docking motif in TRF1 and TRF2 used for differential recruitment of telomeric proteins. Science.

[32] Shibuya H., Ishiguro K., Watanabe Y. (2014). The TRF1-binding protein TERB1 promotes chromosome movement and telomere rigidity in meiosis. Nat. Cell Biol..

[33] Li J.S., Miralles Fuste J., Simavorian T., Bartocci C., Tsai J., Karlseder J., Lazzerini Denchi E. (2017). TZAP: A telomere-associated protein involved in telomere length control. Science.

[34] Wu P., van Overbeek M., Rooney S., de Lange T. (2010). Apollo contributes to G overhang maintenance and protects leading-end telomeres. Mol. Cell.

[35] Lenain C., Bauwens S., Amiard S., Brunori M., Giraud-Panis M.J., Gilson E. (2006). The Apollo 5′ exonuclease functions together with TRF2 to protect telomeres from DNA repair. Curr. Biol..

[36] Rai R., Hu C., Broton C., Chen Y., Lei M., Chang S. (2017). NBS1 Phosphorylation Status Dictates Repair Choice of Dysfunctional Telomeres. Mol. Cell.

[37] Wan B., Yin J., Horvath K., Sarkar J., Chen Y., Wu J., Wan K., Lu J., Gu P., Yu E.Y. (2013). SLX4 assembles a telomere maintenance toolkit by bridging multiple endonucleases with telomeres. Cell Rep..

[38] Lim C.J., Cech T.R. (2021). Shaping human telomeres: From shelterin and CST complexes to telomeric chromatin organization. Nat Rev. Mol. Cell Biol..

[39] Vannier J.B., Pavicic-Kaltenbrunner V., Petalcorin M.I., Ding H., Boulton S.J. (2012). RTEL1 dismantles T loops and counteracts telomeric G4-DNA to maintain telomere integrity. Cell.

[40] Smith S., de Lange T. (2000). Tankyrase promotes telomere elongation in human cells. Curr. Biol..

[41] Cook B.D., Dynek J.N., Chang W., Shostak G., Smith S. (2002). Role for the related poly(ADP-Ribose) polymerases tankyrase 1 and 2 at human telomeres. Mol. Cell. Biol..

[42] Gonzalo S., Garcia-Cao M., Fraga M.F., Schotta G., Peters A.H., Cotter S.E., Eguia R., Dean D.C., Esteller M., Jenuwein T. (2005). Role of the RB1 family in stabilizing histone methylation at constitutive heterochromatin. Nat. Cell Biol..

[43] Garcia-Cao M., O’Sullivan R., Peters A.H., Jenuwein T., Blasco M.A. (2004). Epigenetic regulation of telomere length in mammalian cells by the Suv39h1 and Suv39h2 histone methyltransferases. Nat. Genet..

[44] Dejardin J., Kingston R.E. (2009). Purification of proteins associated with specific genomic Loci. Cell.

[45] Udugama M., FT M.C., Chan F.L., Tang M.C., Pickett H.A., JD R.M., Mayne L., Collas P., Mann J.R., Wong L.H. (2015). Histone variant H3.3 provides the heterochromatic H3 lysine 9 tri-methylation mark at telomeres. Nucleic Acids Res..

[46] Jones B., Su H., Bhat A., Lei H., Bajko J., Hevi S., Baltus G.A., Kadam S., Zhai H., Valdez R. (2008). The histone H3K79 methyltransferase Dot1L is essential for mammalian development and heterochromatin structure. PLoS Genet..

[47] Michishita E., McCord R.A., Berber E., Kioi M., Padilla-Nash H., Damian M., Cheung P., Kusumoto R., Kawahara T.L., Barrett J.C. (2008). SIRT6 is a histone H3 lysine 9 deacetylase that modulates telomeric chromatin. Nature.

[48] Wu Z., Liu J., Zhang Q.D., Lv D.K., Wu N.F., Zhou J.Q. (2017). Rad6-Bre1-mediated H2B ubiquitination regulates telomere replication by promoting telomere-end resection. Nucleic Acids Res..

[49] Ghanim G.E., Fountain A.J., van Roon A.M., Rangan R., Das R., Collins K., Nguyen T.H.D. (2021). Structure of human telomerase holoenzyme with bound telomeric DNA. Nature.

[50] Wright W.E., Piatyszek M.A., Rainey W.E., Byrd W., Shay J.W. (1996). Telomerase activity in human germline and embryonic tissues and cells. Dev. Genet..

[51] Liu L., Bailey S.M., Okuka M., Munoz P., Li C., Zhou L., Wu C., Czerwiec E., Sandler L., Seyfang A. (2007). Telomere lengthening early in development. Nat. Cell Biol..

[52] Allen N.D., Baird D.M. (2009). Telomere length maintenance in stem cell populations. Biochim. Biophys. Acta.

[53] Capper R., Britt-Compton B., Tankimanova M., Rowson J., Letsolo B., Man S., Haughton M., Baird D.M. (2007). The nature of telomere fusion and a definition of the critical telomere length in human cells. Genes Dev..

[54] Counter C.M., Avilion A.A., LeFeuvre C.E., Stewart N.G., Greider C.W., Harley C.B., Bacchetti S. (1992). Telomere shortening associated with chromosome instability is arrested in immortal cells which express telomerase activity. EMBO J..

[55] O’Hagan R.C., Chang S., Maser R.S., Mohan R., Artandi S.E., Chin L., DePinho R.A. (2002). Telomere dysfunction provokes regional amplification and deletion in cancer genomes. Cancer Cell.

[56] Murnane J.P. (2012). Telomere dysfunction and chromosome instability. Mutat. Res..

[57] Chin L., Artandi S.E., Shen Q., Tam A., Lee S.L., Gottlieb G.J., Greider C.W., DePinho R.A. (1999). p53 deficiency rescues the adverse effects of telomere loss and cooperates with telomere dysfunction to accelerate carcinogenesis. Cell.

[58] Barthel F.P., Wei W., Tang M., Martinez-Ledesma E., Hu X., Amin S.B., Akdemir K.C., Seth S., Song X., Wang Q. (2017). Systematic analysis of telomere length and somatic alterations in 31 cancer types. Nat. Genet..

[59] Dilley R.L., Verma P., Cho N.W., Winters H.D., Wondisford A.R., Greenberg R.A. (2016). Break-induced telomere synthesis underlies alternative telomere maintenance. Nature.

[60] Saretzki G. (2014). Extra-telomeric functions of human telomerase: Cancer, mitochondria and oxidative stress. Curr. Pharm. Des..

[61] Podlevsky J.D., Chen J.J. (2012). It all comes together at the ends: Telomerase structure, function, and biogenesis. Mutat. Res..

[62] Venteicher A.S., Abreu E.B., Meng Z., McCann K.E., Terns R.M., Veenstra T.D., Terns M.P., Artandi S.E. (2009). A human telomerase holoenzyme protein required for Cajal body localization and telomere synthesis. Science.

[63] Cong Y.S., Wright W.E., Shay J.W. (2002). Human telomerase and its regulation. Microbiol. Mol. Biol. Rev..

[64] Sharma S., Mukherjee A.K., Roy S.S., Bagri S., Lier S., Verma M., Sengupta A., Kumar M., Nesse G., Pandey D.P. (2021). Human telomerase is directly regulated by non-telomeric TRF2-G-quadruplex interaction. Cell Rep..

[65] Chung S.S., Aroh C., Vadgama J.V. (2013). Constitutive activation of STAT3 signaling regulates hTERT and promotes stem cell-like traits in human breast cancer cells. PLoS ONE.

[66] Khattar E., Tergaonkar V. (2017). Transcriptional Regulation of Telomerase Reverse Transcriptase (TERT) by MYC. Front Cell Dev. Biol..

[67] Yin L., Hubbard A.K., Giardina C. (2000). NF-kappa B regulates transcription of the mouse telomerase catalytic subunit. J. Biol. Chem..

[68] Avin B.A., Umbricht C.B., Zeiger M.A. (2016). Human telomerase reverse transcriptase regulation by DNA methylation, transcription factor binding and alternative splicing (Review). Int. J. Oncol..

[69] Poos A.M., Kordass T., Kolte A., Ast V., Oswald M., Rippe K., Konig R. (2019). Modelling TERT regulation across 19 different cancer types based on the MIPRIP 2.0 gene regulatory network approach. BMC Bioinform..

[70] Wu K.J., Grandori C., Amacker M., Simon-Vermot N., Polack A., Lingner J., Dalla-Favera R. (1999). Direct activation of TERT transcription by c-MYC. Nat. Genet..

[71] Xu D., Popov N., Hou M., Wang Q., Bjorkholm M., Gruber A., Menkel A.R., Henriksson M. (2001). Switch from Myc/Max to Mad1/Max binding and decrease in histone acetylation at the telomerase reverse transcriptase promoter during differentiation of HL60 cells. Proc. Natl. Acad. Sci. USA.

[72] Li Y., Zhou Q.L., Sun W., Chandrasekharan P., Cheng H.S., Ying Z., Lakshmanan M., Raju A., Tenen D.G., Cheng S.Y. (2015). Non-canonical NF-kappaB signalling and ETS1/2 cooperatively drive C250T mutant TERT promoter activation. Nat. Cell Biol..

[73] Konnikova L., Simeone M.C., Kruger M.M., Kotecki M., Cochran B.H. (2005). Signal transducer and activator of transcription 3 (STAT3) regulates human telomerase reverse transcriptase (hTERT) expression in human cancer and primary cells. Cancer Res..

[74] Bowman T., Broome M.A., Sinibaldi D., Wharton W., Pledger W.J., Sedivy J.M., Irby R., Yeatman T., Courtneidge S.A., Jove R. (2001). Stat3-mediated Myc expression is required for Src transformation and PDGF-induced mitogenesis. Proc. Natl. Acad. Sci. USA.

[75] Kidder B.L., Yang J., Palmer S. (2008). Stat3 and c-Myc genome-wide promoter occupancy in embryonic stem cells. PLoS ONE.

[76] Kyo S., Takakura M., Taira T., Kanaya T., Itoh H., Yutsudo M., Ariga H., Inoue M. (2000). Sp1 cooperates with c-Myc to activate transcription of the human telomerase reverse transcriptase gene (hTERT). Nucleic Acids Res..

[77] Beishline K., Azizkhan-Clifford J. (2015). Sp1 and the ‘hallmarks of cancer’. FEBS J..

[78] Bermudez Y., Yang H., Cheng J.Q., Kruk P.A. (2008). Pyk2/ERK 1/2 mediate Sp1- and c-Myc-dependent induction of telomerase activity by epidermal growth factor. Growth Factors.

[79] Pore N., Liu S., Shu H.K., Li B., Haas-Kogan D., Stokoe D., Milanini-Mongiat J., Pages G., O’Rourke D.M., Bernhard E. (2004). Sp1 is involved in Akt-mediated induction of VEGF expression through an HIF-1-independent mechanism. Mol. Biol. Cell.

[80] Baudino T.A., McKay C., Pendeville-Samain H., Nilsson J.A., Maclean K.H., White E.L., Davis A.C., Ihle J.N., Cleveland J.L. (2002). c-Myc is essential for vasculogenesis and angiogenesis during development and tumor progression. Genes Dev..

[81] Jungert K., Buck A., von Wichert G., Adler G., Konig A., Buchholz M., Gress T.M., Ellenrieder V. (2007). Sp1 is required for transforming growth factor-beta-induced mesenchymal transition and migration in pancreatic cancer cells. Cancer Res..

[82] Cowling V.H., D’Cruz C.M., Chodosh L.A., Cole M.D. (2007). c-Myc transforms human mammary epithelial cells through repression of the Wnt inhibitors DKK1 and SFRP1. Mol. Cell. Biol..

[83] Rennoll S., Yochum G. (2015). Regulation of MYC gene expression by aberrant Wnt/beta-catenin signaling in colorectal cancer. World J. Biol. Chem..

[84] Mir R., Sharma A., Pradhan S.J., Galande S. (2018). Regulation of Transcription Factor SP1 by the beta-Catenin Destruction Complex Modulates Wnt Response. Mol. Cell. Biol..

[85] Lorbeer F.K., Hockemeyer D. (2020). TERT promoter mutations and telomeres during tumorigenesis. Curr. Opin. Genet. Dev..

[86] Hafezi F., Perez Bercoff D. (2020). The Solo Play of TERT Promoter Mutations. Cells.

[87] Liu R., Zhang T., Zhu G., Xing M. (2018). Regulation of mutant TERT by BRAF V600E/MAP kinase pathway through FOS/GABP in human cancer. Nat. Commun..

[88] Song Y.S., Yoo S.K., Kim H.H., Jung G., Oh A.R., Cha J.Y., Kim S.J., Cho S.W., Lee K.E., Seo J.S. (2019). Interaction of BRAF-induced ETS factors with mutant TERT promoter in papillary thyroid cancer. Endocr. Relat. Cancer.

[89] Oh S., Song Y.H., Yim J., Kim T.K. (2000). Identification of Mad as a repressor of the human telomerase (hTERT) gene. Oncogene.

[90] Kanaya T., Kyo S., Hamada K., Takakura M., Kitagawa Y., Harada H., Inoue M. (2000). Adenoviral expression of p53 represses telomerase activity through down-regulation of human telomerase reverse transcriptase transcription. Clin. Cancer Res. Off. J. Am. Assoc. Cancer Res..

[91] Xu D., Wang Q., Gruber A., Bjorkholm M., Chen Z., Zaid A., Selivanova G., Peterson C., Wiman K.G., Pisa P. (2000). Downregulation of telomerase reverse transcriptase mRNA expression by wild type p53 in human tumor cells. Oncogene.

[92] Renaud S., Loukinov D., Bosman F.T., Lobanenkov V., Benhattar J. (2005). CTCF binds the proximal exonic region of hTERT and inhibits its transcription. Nucleic Acids Res..

[93] Renaud S., Loukinov D., Abdullaev Z., Guilleret I., Bosman F.T., Lobanenkov V., Benhattar J. (2007). Dual role of DNA methylation inside and outside of CTCF-binding regions in the transcriptional regulation of the telomerase hTERT gene. Nucleic Acids Res..

[94] Yuan X., Larsson C., Xu D. (2019). Mechanisms underlying the activation of TERT transcription and telomerase activity in human cancer: Old actors and new players. Oncogene.

[95] Liu C., Fang X., Ge Z., Jalink M., Kyo S., Bjorkholm M., Gruber A., Sjoberg J., Xu D. (2007). The telomerase reverse transcriptase (hTERT) gene is a direct target of the histone methyltransferase SMYD3. Cancer Res..

[96] Thakur R.K., Yadav V.K., Kumar P., Chowdhury S. (2011). Mechanisms of non-metastatic 2 (NME2)-mediated control of metastasis across tumor types. Naunyn. Schmiedebergs Arch. Pharm..

[97] Saha D., Singh A., Hussain T., Srivastava V., Sengupta S., Kar A., Dhapola P., Dhople V., Ummanni R., Chowdhury S. (2017). Epigenetic suppression of human telomerase (hTERT) is mediated by the metastasis suppressor NME2 in a G-quadruplex-dependent fashion. J. Biol. Chem..

[98] Kar A., Saha D., Purohit G., Singh A., Kumar P., Yadav V.K., Kumar P., Thakur R.K., Chowdhury S. (2012). Metastases suppressor NME2 associates with telomere ends and telomerase and reduces telomerase activity within cells. Nucleic Acids Res..

[99] Guilleret I., Yan P., Grange F., Braunschweig R., Bosman F.T., Benhattar J. (2002). Hypermethylation of the human telomerase catalytic subunit (hTERT) gene correlates with telomerase activity. Int. J. Cancer.

[100] Saeboe-Larssen S., Fossberg E., Gaudernack G. (2006). Characterization of novel alternative splicing sites in human telomerase reverse transcriptase (hTERT): Analysis of expression and mutual correlation in mRNA isoforms from normal and tumour tissues. BMC Mol. Biol..

[101] Yi X., White D.M., Aisner D.L., Baur J.A., Wright W.E., Shay J.W. (2000). An alternate splicing variant of the human telomerase catalytic subunit inhibits telomerase activity. Neoplasia.

[102] Listerman I., Sun J., Gazzaniga F.S., Lukas J.L., Blackburn E.H. (2013). The major reverse transcriptase-incompetent splice variant of the human telomerase protein inhibits telomerase activity but protects from apoptosis. Cancer Res..

[103] Colgin L.M., Wilkinson C., Englezou A., Kilian A., Robinson M.O., Reddel R.R. (2000). The hTERTalpha splice variant is a dominant negative inhibitor of telomerase activity. Neoplasia.

[104] Cerezo A., Kalthoff H., Schuermann M., Schafer B., Boukamp P. (2002). Dual regulation of telomerase activity through c-Myc-dependent inhibition and alternative splicing of hTERT. J. Cell Sci..

[105] Song G., Wang R., Guo J., Liu X., Wang F., Qi Y., Wan H., Liu M., Li X., Tang H. (2015). miR-346 and miR-138 competitively regulate hTERT in GRSF1- and AGO2-dependent manners, respectively. Sci. Rep..

[106] Ohira T., Naohiro S., Nakayama Y., Osaki M., Okada F., Oshimura M., Kugoh H. (2015). miR-19b regulates hTERT mRNA expression through targeting PITX1 mRNA in melanoma cells. Sci. Rep..

[107] Lu M.H., Tang B., Zeng S., Hu C.J., Xie R., Wu Y.Y., Wang S.M., He F.T., Yang S.M. (2016). Long noncoding RNA BC032469, a novel competing endogenous RNA, upregulates hTERT expression by sponging miR-1207-5p and promotes proliferation in gastric cancer. Oncogene.

[108] Zhang X.L., Xu L.L., Wang F. (2017). Hsa_circ_0020397 regulates colorectal cancer cell viability, apoptosis and invasion by promoting the expression of the miR-138 targets TERT and PD-L1. Cell Biol. Int..

[109] Azzalin C.M., Reichenbach P., Khoriauli L., Giulotto E., Lingner J. (2007). Telomeric repeat containing RNA and RNA surveillance factors at mammalian chromosome ends. Science.

[110] Redon S., Reichenbach P., Lingner J. (2010). The non-coding RNA TERRA is a natural ligand and direct inhibitor of human telomerase. Nucleic Acids Res..

[111] Fernandes R.V., Feretzaki M., Lingner J. (2021). The makings of TERRA R-loops at chromosome ends. Cell Cycle.

[112] Montero J.J., Lopez-Silanes I., Megias D., Fraga M.F., Castells-Garcia A., Blasco M.A. (2018). TERRA recruitment of polycomb to telomeres is essential for histone trymethylation marks at telomeric heterochromatin. Nat. Commun..

[113] Chow T.T., Shi X., Wei J.H., Guan J., Stadler G., Huang B., Blackburn E.H. (2018). Local enrichment of HP1alpha at telomeres alters their structure and regulation of telomere protection. Nat. Commun..

[114] Arora R., Lee Y., Wischnewski H., Brun C.M., Schwarz T., Azzalin C.M. (2014). RNaseH1 regulates TERRA-telomeric DNA hybrids and telomere maintenance in ALT tumour cells. Nat. Commun..

[115] Yi X., Tesmer V.M., Savre-Train I., Shay J.W., Wright W.E. (1999). Both transcriptional and posttranscriptional mechanisms regulate human telomerase template RNA levels. Mol. Cell. Biol..

[116] Goldfarb K.C., Cech T.R. (2013). 3’ terminal diversity of MRP RNA and other human noncoding RNAs revealed by deep sequencing. BMC Mol. Biol..

[117] Tseng C.K., Wang H.F., Schroeder M.R., Baumann P. (2018). The H/ACA complex disrupts triplex in hTR precursor to permit processing by RRP6 and PARN. Nat. Commun..

[118] Kim N.K., Theimer C.A., Mitchell J.R., Collins K., Feigon J. (2010). Effect of pseudouridylation on the structure and activity of the catalytically essential P6.1 hairpin in human telomerase RNA. Nucleic Acids Res..

[119] Tseng C.K., Wang H.F., Burns A.M., Schroeder M.R., Gaspari M., Baumann P. (2015). Human Telomerase RNA Processing and Quality Control. Cell Rep..

[120] Moon D.H., Segal M., Boyraz B., Guinan E., Hofmann I., Cahan P., Tai A.K., Agarwal S. (2015). Poly(A)-specific ribonuclease (PARN) mediates 3′-end maturation of the telomerase RNA component. Nat. Genet.

[121] Roake C.M., Chen L., Chakravarthy A.L., Ferrell J.E., Raffa G.D., Artandi S.E. (2019). Disruption of Telomerase RNA Maturation Kinetics Precipitates Disease. Mol. Cell.

[122] Shukla S., Schmidt J.C., Goldfarb K.C., Cech T.R., Parker R. (2016). Inhibition of telomerase RNA decay rescues telomerase deficiency caused by dyskerin or PARN defects. Nat. Struct. Mol. Biol..

[123] Tang W., Kannan R., Blanchette M., Baumann P. (2012). Telomerase RNA biogenesis involves sequential binding by Sm and Lsm complexes. Nature.

[124] Chen L., Roake C.M., Galati A., Bavasso F., Micheli E., Saggio I., Schoeftner S., Cacchione S., Gatti M., Artandi S.E. (2020). Loss of Human TGS1 Hypermethylase Promotes Increased Telomerase RNA and Telomere Elongation. Cell Rep..

[125] Savelyev N.V., Shepelev N.M., Lavrik O.I., Rubtsova M.P., Dontsova O.A. (2021). PARP1 Regulates the Biogenesis and Activity of Telomerase Complex Through Modification of H/ACA-Proteins. Front. Cell Dev. Biol..

[126] Beneke S., Cohausz O., Malanga M., Boukamp P., Althaus F., Burkle A. (2008). Rapid regulation of telomere length is mediated by poly(ADP-ribose) polymerase-1. Nucleic Acids Res..

[127] Hamma T., Ferre-D’Amare A.R. (2010). The box H/ACA ribonucleoprotein complex: Interplay of RNA and protein structures in post-transcriptional RNA modification. J. Biol. Chem..

[128] Stanek D., Pridalova-Hnilicova J., Novotny I., Huranova M., Blazikova M., Wen X., Sapra A.K., Neugebauer K.M. (2008). Spliceosomal small nuclear ribonucleoprotein particles repeatedly cycle through Cajal bodies. Mol. Biol. Cell.

[129] Mahmoudi S., Henriksson S., Weibrecht I., Smith S., Soderberg O., Stromblad S., Wiman K.G., Farnebo M. (2010). WRAP53 is essential for Cajal body formation and for targeting the survival of motor neuron complex to Cajal bodies. PLoS Biol..

[130] Wang Q., Sawyer I.A., Sung M.H., Sturgill D., Shevtsov S.P., Pegoraro G., Hakim O., Baek S., Hager G.L., Dundr M. (2016). Cajal bodies are linked to genome conformation. Nat. Commun..

[131] Gu P., Jia S., Takasugi T., Tesmer V.M., Nandakumar J., Chen Y., Chang S. (2021). Distinct functions of POT1 proteins contribute to the regulation of telomerase recruitment to telomeres. Nat. Commun..

[132] Wang F., Podell E.R., Zaug A.J., Yang Y., Baciu P., Cech T.R., Lei M. (2007). The POT1-TPP1 telomere complex is a telomerase processivity factor. Nature.

[133] Nandakumar J., Bell C.F., Weidenfeld I., Zaug A.J., Leinwand L.A., Cech T.R. (2012). The TEL patch of telomere protein TPP1 mediates telomerase recruitment and processivity. Nature.

[134] Frank A.K., Tran D.C., Qu R.W., Stohr B.A., Segal D.J., Xu L. (2015). The Shelterin TIN2 Subunit Mediates Recruitment of Telomerase to Telomeres. PLoS Genet..

[135] Greider C.W. (2016). Regulating telomere length from the inside out: The replication fork model. Genes Dev..

[136] Xu M., Axhemi A., Malgowska M., Chen Y., Leonard D., Srinivasan S., Jankowsky E., Taylor D.J. (2021). Active and Passive Destabilization of G-Quadruplex DNA by the Telomere POT1-TPP1 Complex. J. Mol. Biol..

[137] Walker J.R., Zhu X.D. (2012). Post-translational modifications of TRF1 and TRF2 and their roles in telomere maintenance. Mech. Ageing Dev..

[138] Smith L.L., Coller H.A., Roberts J.M. (2003). Telomerase modulates expression of growth-controlling genes and enhances cell proliferation. Nat. Cell Biol..

[139] Liu N., Ding D., Hao W., Yang F., Wu X., Wang M., Xu X., Ju Z., Liu J.P., Song Z. (2016). hTERT promotes tumor angiogenesis by activating VEGF via interactions with the Sp1 transcription factor. Nucleic Acids Res..

[140] Zaccagnini G., Gaetano C., Della Pietra L., Nanni S., Grasselli A., Mangoni A., Benvenuto R., Fabrizi M., Truffa S., Germani A. (2005). Telomerase mediates vascular endothelial growth factor-dependent responsiveness in a rat model of hind limb ischemia. J. Biol. Chem..

[141] Coleman C., Levine D., Kishore R., Qin G., Thorne T., Lambers E., Sasi S.P., Yaar M., Gilchrest B.A., Goukassian D.A. (2010). Inhibition of melanoma angiogenesis by telomere homolog oligonucleotides. J. Oncol..

[142] Pallini R., Sorrentino A., Pierconti F., Maggiano N., Faggi R., Montano N., Maira G., Larocca L.M., Levi A., Falchetti M.L. (2006). Telomerase inhibition by stable RNA interference impairs tumor growth and angiogenesis in glioblastoma xenografts. Int. J. Cancer.

[143] Zhang X., Gaspard J.P., Chung D.C. (2001). Regulation of vascular endothelial growth factor by the Wnt and K-ras pathways in colonic neoplasia. Cancer Res..

[144] He T.C., Sparks A.B., Rago C., Hermeking H., Zawel L., da Costa L.T., Morin P.J., Vogelstein B., Kinzler K.W. (1998). Identification of c-MYC as a target of the APC pathway. Science.

[145] Park J.I., Venteicher A.S., Hong J.Y., Choi J., Jun S., Shkreli M., Chang W., Meng Z., Cheung P., Ji H. (2009). Telomerase modulates Wnt signalling by association with target gene chromatin. Nature.

[146] Hoffmeyer K., Raggioli A., Rudloff S., Anton R., Hierholzer A., Del Valle I., Hein K., Vogt R., Kemler R. (2012). Wnt/beta-catenin signaling regulates telomerase in stem cells and cancer cells. Science.

[147] Ding D., Xi P., Zhou J., Wang M., Cong Y.S. (2013). Human telomerase reverse transcriptase regulates MMP expression independently of telomerase activity via NF-kappaB-dependent transcription. FASEB J. Off. Publ. Fed. Am. Soc. Exp. Biol..

[148] Huber M.A., Azoitei N., Baumann B., Grunert S., Sommer A., Pehamberger H., Kraut N., Beug H., Wirth T. (2004). NF-kappaB is essential for epithelial-mesenchymal transition and metastasis in a model of breast cancer progression. J. Clin. Investig..

[149] Qin Y., Tang B., Hu C.J., Xiao Y.F., Xie R., Yong X., Wu Y.Y., Dong H., Yang S.M. (2016). An hTERT/ZEB1 complex directly regulates E-cadherin to promote epithelial-to-mesenchymal transition (EMT) in colorectal cancer. Oncotarget.

[150] Akiyama M., Hideshima T., Hayashi T., Tai Y.T., Mitsiades C.S., Mitsiades N., Chauhan D., Richardson P., Munshi N.C., Anderson K.C. (2002). Cytokines modulate telomerase activity in a human multiple myeloma cell line. Cancer Res..

[151] Zhan T., Rindtorff N., Boutros M. (2017). Wnt signaling in cancer. Oncogene.

[152] Faubert B., Solmonson A., DeBerardinis R.J. (2020). Metabolic reprogramming and cancer progression. Science.

[153] Porporato P.E., Filigheddu N., Pedro J.M.B., Kroemer G., Galluzzi L. (2018). Mitochondrial metabolism and cancer. Cell Res..

[154] Haendeler J., Drose S., Buchner N., Jakob S., Altschmied J., Goy C., Spyridopoulos I., Zeiher A.M., Brandt U., Dimmeler S. (2009). Mitochondrial telomerase reverse transcriptase binds to and protects mitochondrial DNA and function from damage. Arter. Thromb. Vasc. Biol..

[155] Ahmed S., Passos J.F., Birket M.J., Beckmann T., Brings S., Peters H., Birch-Machin M.A., von Zglinicki T., Saretzki G. (2008). Telomerase does not counteract telomere shortening but protects mitochondrial function under oxidative stress. J. Cell Sci..

[156] Sahin E., Colla S., Liesa M., Moslehi J., Muller F.L., Guo M., Cooper M., Kotton D., Fabian A.J., Walkey C. (2011). Telomere dysfunction induces metabolic and mitochondrial compromise. Nature.

[157] Ale-Agha N., Jakobs P., Goy C., Zurek M., Rosen J., Dyballa-Rukes N., Metzger S., Greulich J., von Ameln F., Eckermann O. (2021). Mitochondrial Telomerase Reverse Transcriptase Protects From Myocardial Ischemia/Reperfusion Injury by Improving Complex I Composition and Function. Circulation.

[158] Martens A., Schmid B., Akintola O., Saretzki G. (2019). Telomerase Does Not Improve DNA Repair in Mitochondria upon Stress but Increases MnSOD Protein under Serum-Free Conditions. Int. J. Mol. Sci..

[159] Indran I.R., Hande M.P., Pervaiz S. (2011). hTERT overexpression alleviates intracellular ROS production, improves mitochondrial function, and inhibits ROS-mediated apoptosis in cancer cells. Cancer Res..

[160] Nishikawa M. (2008). Reactive oxygen species in tumor metastasis. Cancer Lett..

[161] Piskounova E., Agathocleous M., Murphy M.M., Hu Z., Huddlestun S.E., Zhao Z., Leitch A.M., Johnson T.M., DeBerardinis R.J., Morrison S.J. (2015). Oxidative stress inhibits distant metastasis by human melanoma cells. Nature.

[162] Ahmad F., Patrick S., Sheikh T., Sharma V., Pathak P., Malgulwar P.B., Kumar A., Joshi S.D., Sarkar C., Sen E. (2017). Telomerase reverse transcriptase (TERT)—enhancer of zeste homolog 2 (EZH2) network regulates lipid metabolism and DNA damage responses in glioblastoma. J. Neurochem..

[163] Shaheen F., Grammatopoulos D.K., Muller J., Zammit V.A., Lehnert H. (2014). Extra-nuclear telomerase reverse transcriptase (TERT) regulates glucose transport in skeletal muscle cells. Biochim. Biophys. Acta.

[164] Viswanath P., Batsios G., Ayyappan V., Taglang C., Gillespie A.M., Larson P.E.Z., Luchman H.A., Costello J.F., Pieper R.O., Ronen S.M. (2021). Metabolic imaging detects elevated glucose flux through the pentose phosphate pathway associated with TERT expression in low-grade gliomas. Neuro-Oncology.

[165] Lamb R., Ozsvari B., Bonuccelli G., Smith D.L., Pestell R.G., Martinez-Outschoorn U.E., Clarke R.B., Sotgia F., Lisanti M.P. (2015). Dissecting tumor metabolic heterogeneity: Telomerase and large cell size metabolically define a sub-population of stem-like, mitochondrial-rich, cancer cells. Oncotarget.

[166] O’Sullivan R.J., Karlseder J. (2010). Telomeres: Protecting chromosomes against genome instability. Nat. Rev. Mol. Cell Biol..

[167] Ducray C., Pommier J.P., Martins L., Boussin F.D., Sabatier L. (1999). Telomere dynamics, end-to-end fusions and telomerase activation during the human fibroblast immortalization process. Oncogene.

[168] Suram A., Kaplunov J., Patel P.L., Ruan H., Cerutti A., Boccardi V., Fumagalli M., Di Micco R., Mirani N., Gurung R.L. (2012). Oncogene-induced telomere dysfunction enforces cellular senescence in human cancer precursor lesions. EMBO J..

[169] Meena J.K., Cerutti A., Beichler C., Morita Y., Bruhn C., Kumar M., Kraus J.M., Speicher M.R., Wang Z.Q., Kestler H.A. (2015). Telomerase abrogates aneuploidy-induced telomere replication stress, senescence and cell depletion. EMBO J..

[170] Matmati S., Lambert S., Geli V., Coulon S. (2020). Telomerase Repairs Collapsed Replication Forks at Telomeres. Cell Rep..

[171] Blasco M.A., Rizen M., Greider C.W., Hanahan D. (1996). Differential regulation of telomerase activity and telomerase RNA during multi-stage tumorigenesis. Nat. Genet..

[172] Chu C., Qu K., Zhong F.L., Artandi S.E., Chang H.Y. (2011). Genomic maps of long noncoding RNA occupancy reveal principles of RNA-chromatin interactions. Mol. Cell.

[173] Liu H., Yang Y., Ge Y., Liu J., Zhao Y. (2019). TERC promotes cellular inflammatory response independent of telomerase. Nucleic Acids Res..

[174] Cheng Y., Liu P., Zheng Q., Gao G., Yuan J., Wang P., Huang J., Xie L., Lu X., Tong T. (2018). Mitochondrial Trafficking and Processing of Telomerase RNA TERC. Cell Rep..

[175] Rubtsova M., Naraykina Y., Vasilkova D., Meerson M., Zvereva M., Prassolov V., Lazarev V., Manuvera V., Kovalchuk S., Anikanov N. (2018). Protein encoded in human telomerase RNA is involved in cell protective pathways. Nucleic Acids Res..

[176] Gazzaniga F.S., Blackburn E.H. (2014). An antiapoptotic role for telomerase RNA in human immune cells independent of telomere integrity or telomerase enzymatic activity. Blood.

[177] Kroupa M., Rachakonda S.K., Liska V., Srinivas N., Urbanova M., Jiraskova K., Schneiderova M., Vycital O., Vymetalkova V., Vodickova L. (2019). Relationship of telomere length in colorectal cancer patients with cancer phenotype and patient prognosis. Br. J. Cancer.

[178] Zhang C., Chen X., Li L., Zhou Y., Wang C., Hou S. (2015). The Association between Telomere Length and Cancer Prognosis: Evidence from a Meta-Analysis. PLoS ONE.

[179] Ennour-Idrissi K., Maunsell E., Diorio C. (2017). Telomere Length and Breast Cancer Prognosis: A Systematic Review. Cancer Epidemiol. Biomark. Prev..

[180] Oh B.K., Kim H., Park Y.N., Yoo J.E., Choi J., Kim K.S., Lee J.J., Park C. (2008). High telomerase activity and long telomeres in advanced hepatocellular carcinomas with poor prognosis. Lab. Investig..

[181] Gallicchio L., Gadalla S.M., Murphy J.D., Simonds N.I. (2018). The Effect of Cancer Treatments on Telomere Length: A Systematic Review of the Literature. J. Natl. Cancer Inst..

[182] Heaphy C.M., Subhawong A.P., Hong S.M., Goggins M.G., Montgomery E.A., Gabrielson E., Netto G.J., Epstein J.I., Lotan T.L., Westra W.H. (2011). Prevalence of the alternative lengthening of telomeres telomere maintenance mechanism in human cancer subtypes. Am. J. Pathol..

[183] Subhawong A.P., Heaphy C.M., Argani P., Konishi Y., Kouprina N., Nassar H., Vang R., Meeker A.K. (2009). The alternative lengthening of telomeres phenotype in breast carcinoma is associated with HER-2 overexpression. Mod. Pathol. Off. J. U. S. Can. Acad. Pathol. Inc..

[184] Sampl S., Pramhas S., Stern C., Preusser M., Marosi C., Holzmann K. (2012). Expression of telomeres in astrocytoma WHO grade 2 to 4: TERRA level correlates with telomere length, telomerase activity, and advanced clinical grade. Transl. Oncol..

[185] Vitelli V., Falvo P., Nergadze S.G., Santagostino M., Khoriauli L., Pellanda P., Bertino G., Occhini A., Benazzo M., Morbini P. (2018). Telomeric Repeat-Containing RNAs (TERRA) Decrease in Squamous Cell Carcinoma of the Head and Neck Is Associated with Worsened Clinical Outcome. Int. J. Mol. Sci..

[186] Cao H., Zhai Y., Ji X., Wang Y., Zhao J., Xing J., An J., Ren T. (2020). Noncoding telomeric repeat-containing RNA inhibits the progression of hepatocellular carcinoma by regulating telomerase-mediated telomere length. Cancer Sci..

[187] Leao R., Apolonio J.D., Lee D., Figueiredo A., Tabori U., Castelo-Branco P. (2018). Mechanisms of human telomerase reverse transcriptase (hTERT) regulation: Clinical impacts in cancer. J. Biomed. Sci..

[188] Bell R.J., Rube H.T., Kreig A., Mancini A., Fouse S.D., Nagarajan R.P., Choi S., Hong C., He D., Pekmezci M. (2015). The transcription factor GABP selectively binds and activates the mutant TERT promoter in cancer. Science.

[189] Robinson N.J., Schiemann W.P. (2016). Means to the ends: The role of telomeres and telomere processing machinery in metastasis. Biochim. Biophys. Acta.

[190] Hochreiter A.E., Xiao H., Goldblatt E.M., Gryaznov S.M., Miller K.D., Badve S., Sledge G.W., Herbert B.S. (2006). Telomerase template antagonist GRN163L disrupts telomere maintenance, tumor growth, and metastasis of breast cancer. Clin. Cancer Res. Off. J. Am. Assoc. Cancer Res..

[191] Dikmen Z.G., Gellert G.C., Jackson S., Gryaznov S., Tressler R., Dogan P., Wright W.E., Shay J.W. (2005). In vivo inhibition of lung cancer by GRN163L: A novel human telomerase inhibitor. Cancer Res..

[192] Hernandez-Sanchez W., Huang W., Plucinsky B., Garcia-Vazquez N., Robinson N.J., Schiemann W.P., Berdis A.J., Skordalakes E., Taylor D.J. (2019). A non-natural nucleotide uses a specific pocket to selectively inhibit telomerase activity. PLoS Biol..

[193] Jafri M.A., Ansari S.A., Alqahtani M.H., Shay J.W. (2016). Roles of telomeres and telomerase in cancer, and advances in telomerase-targeted therapies. Genome Med..

[194] Chiappori A.A., Kolevska T., Spigel D.R., Hager S., Rarick M., Gadgeel S., Blais N., Von Pawel J., Hart L., Reck M. (2015). A randomized phase II study of the telomerase inhibitor imetelstat as maintenance therapy for advanced non-small-cell lung cancer. Ann. Oncol..

[195] Queisser A., Heeg S., Thaler M., von Werder A., Opitz O.G. (2013). Inhibition of telomerase induces alternative lengthening of telomeres during human esophageal carcinogenesis. Cancer Genet..

[196] Hu J., Hwang S.S., Liesa M., Gan B., Sahin E., Jaskelioff M., Ding Z., Ying H., Boutin A.T., Zhang H. (2012). Antitelomerase therapy provokes ALT and mitochondrial adaptive mechanisms in cancer. Cell.

[197] Gan Y., Mo Y., Johnston J., Lu J., Wientjes M.G., Au J.L. (2002). Telomere maintenance in telomerase-positive human ovarian SKOV-3 cells cannot be retarded by complete inhibition of telomerase. FEBS Lett..

[198] Robinson N.J., Morrison-Smith C.D., Gooding A.J., Schiemann B.J., Jackson M.W., Taylor D.J., Schiemann W.P. (2020). SLX4IP and telomere dynamics dictate breast cancer metastasis and therapeutic responsiveness. Life Sci. Alliance.

[199] Griffith J.K., Bryant J.E., Fordyce C.A., Gilliland F.D., Joste N.E., Moyzis R.K. (1999). Reduced telomere DNA content is correlated with genomic instability and metastasis in invasive human breast carcinoma. Breast Cancer Res. Treat..

[200] Seimiya H., Muramatsu Y., Ohishi T., Tsuruo T. (2005). Tankyrase 1 as a target for telomere-directed molecular cancer therapeutics. Cancer Cell.

[201] Tian X.H., Hou W.J., Fang Y., Fan J., Tong H., Bai S.L., Chen Q., Xu H., Li Y. (2013). XAV939, a tankyrase 1 inhibitior, promotes cell apoptosis in neuroblastoma cell lines by inhibiting Wnt/beta-catenin signaling pathway. J. Exp. Clin. Cancer Res..

[202] Waaler J., Machon O., Tumova L., Dinh H., Korinek V., Wilson S.R., Paulsen J.E., Pedersen N.M., Eide T.J., Machonova O. (2012). A novel tankyrase inhibitor decreases canonical Wnt signaling in colon carcinoma cells and reduces tumor growth in conditional APC mutant mice. Cancer Res..

[203] Zeng X., Hernandez-Sanchez W., Xu M., Whited T.L., Baus D., Zhang J., Berdis A.J., Taylor D.J. (2018). Administration of a Nucleoside Analog Promotes Cancer Cell Death in a Telomerase-Dependent Manner. Cell Rep..

[204] Li S., Rosenberg J.E., Donjacour A.A., Botchkina I.L., Hom Y.K., Cunha G.R., Blackburn E.H. (2004). Rapid inhibition of cancer cell growth induced by lentiviral delivery and expression of mutant-template telomerase RNA and anti-telomerase short-interfering RNA. Cancer Res..

[205] Cerone M.A., Londono-Vallejo J.A., Autexier C. (2006). Mutated telomeres sensitize tumor cells to anticancer drugs independently of telomere shortening and mechanisms of telomere maintenance. Oncogene.

[206] Negrini S., De Palma R., Filaci G. (2020). Anti-cancer Immunotherapies Targeting Telomerase. Cancers.

[207] Nava-Parada P., Emens L.A. (2007). GV-1001, an injectable telomerase peptide vaccine for the treatment of solid cancers. Curr. Opin. Mol..

[208] Fenoglio D., Parodi A., Lavieri R., Kalli F., Ferrera F., Tagliamacco A., Guastalla A., Lamperti M.G., Giacomini M., Filaci G. (2015). Immunogenicity of GX301 cancer vaccine: Four (telomerase peptides) are better than one. Hum. Vaccines Immunother..

[209] Vetsika E.K., Konsolakis G., Aggouraki D., Kotsakis A., Papadimitraki E., Christou S., Menez-Jamet J., Kosmatopoulos K., Georgoulias V., Mavroudis D. (2012). Immunological responses in cancer patients after vaccination with the therapeutic telomerase-specific vaccine Vx-001. Cancer Immunol. Immunother..

[210] Patente T.A., Pinho M.P., Oliveira A.A., Evangelista G.C.M., Bergami-Santos P.C., Barbuto J.A.M. (2018). Human Dendritic Cells: Their Heterogeneity and Clinical Application Potential in Cancer Immunotherapy. Front. Immunol..

[211] Nair S.K., Heiser A., Boczkowski D., Majumdar A., Naoe M., Lebkowski J.S., Vieweg J., Gilboa E. (2000). Induction of cytotoxic T cell responses and tumor immunity against unrelated tumors using telomerase reverse transcriptase RNA transfected dendritic cells. Nat. Med..

[212] Su Z., Dannull J., Yang B.K., Dahm P., Coleman D., Yancey D., Sichi S., Niedzwiecki D., Boczkowski D., Gilboa E. (2005). Telomerase mRNA-transfected dendritic cells stimulate antigen-specific CD8+ and CD4+ T cell responses in patients with metastatic prostate cancer. J. Immunol..

[213] Berntsen A., Trepiakas R., Wenandy L., Geertsen P.F., thor Straten P., Andersen M.H., Pedersen A.E., Claesson M.H., Lorentzen T., Johansen J.S. (2008). Therapeutic dendritic cell vaccination of patients with metastatic renal cell carcinoma: A clinical phase 1/2 trial. J. Immunother..

[214] Vik-Mo E.O., Nyakas M., Mikkelsen B.V., Moe M.C., Due-Tonnesen P., Suso E.M., Saeboe-Larssen S., Sandberg C., Brinchmann J.E., Helseth E. (2013). Therapeutic vaccination against autologous cancer stem cells with mRNA-transfected dendritic cells in patients with glioblastoma. Cancer Immunol. Immunother..

[215] Khoury H.J., Collins R.H., Blum W., Stiff P.S., Elias L., Lebkowski J.S., Reddy A., Nishimoto K.P., Sen D., Wirth E.D. (2017). Immune responses and long-term disease recurrence status after telomerase-based dendritic cell immunotherapy in patients with acute myeloid leukemia. Cancer.

[216] Lilleby W., Gaudernack G., Brunsvig P.F., Vlatkovic L., Schulz M., Mills K., Hole K.H., Inderberg E.M. (2017). Phase I/IIa clinical trial of a novel hTERT peptide vaccine in men with metastatic hormone-naive prostate cancer. Cancer Immunol. Immunother..

[217] Middleton G., Silcocks P., Cox T., Valle J., Wadsley J., Propper D., Coxon F., Ross P., Madhusudan S., Roques T. (2014). Gemcitabine and capecitabine with or without telomerase peptide vaccine GV1001 in patients with locally advanced or metastatic pancreatic cancer (TeloVac): An open-label, randomised, phase 3 trial. Lancet. Oncol..

[218] Thalmensi J., Pliquet E., Liard C., Escande M., Bestetti T., Julithe M., Kostrzak A., Pailhes-Jimenez A.S., Bourges E., Loustau M. (2016). Anticancer DNA vaccine based on human telomerase reverse transcriptase generates a strong and specific T cell immune response. Oncoimmunology.

[219] Teixeira L., Medioni J., Garibal J., Adotevi O., Doucet L., Durey M.D., Ghrieb Z., Kiladjian J.J., Brizard M., Laheurte C. (2020). A First-in-Human Phase I Study of INVAC-1, an Optimized Human Telomerase DNA Vaccine in Patients with Advanced Solid Tumors. Clin. Cancer Res. Off. J. Am. Assoc. Cancer Res..

[220] Sandri S., Bobisse S., Moxley K., Lamolinara A., De Sanctis F., Boschi F., Sbarbati A., Fracasso G., Ferrarini G., Hendriks R.W. (2016). Feasibility of Telomerase-Specific Adoptive T-cell Therapy for B-cell Chronic Lymphocytic Leukemia and Solid Malignancies. Cancer Res..

[221] Hiyama K., Hirai Y., Kyoizumi S., Akiyama M., Hiyama E., Piatyszek M.A., Shay J.W., Ishioka S., Yamakido M. (1995). Activation of telomerase in human lymphocytes and hematopoietic progenitor cells. J. Immunol..

[222] Hiyama E., Tatsumoto N., Kodama T., Hiyama K., Shay J., Yokoyama T. (1996). Telomerase activity in human intestine. Int. J. Oncol..

[223] Pech M.F., Garbuzov A., Hasegawa K., Sukhwani M., Zhang R.J., Benayoun B.A., Brockman S.A., Lin S., Brunet A., Orwig K.E. (2015). High telomerase is a hallmark of undifferentiated spermatogonia and is required for maintenance of male germline stem cells. Genes Dev..

[224] Robinson N.J., Taylor D.J., Schiemann W.P. (2019). Stem cells, immortality, and the evolution of metastatic properties in breast cancer: Telomere maintenance mechanisms and metastatic evolution. J. Cancer Metastasis Treat..

[225] Sotillo-Pineiro E., Sierrasesumaga L., Patinno-Garcia A. (2004). Telomerase activity and telomere length in primary and metastatic tumors from pediatric bone cancer patients. Pediatr. Res..

[226] Flynn R.L., Cox K.E., Jeitany M., Wakimoto H., Bryll A.R., Ganem N.J., Bersani F., Pineda J.R., Suva M.L., Benes C.H. (2015). Alternative lengthening of telomeres renders cancer cells hypersensitive to ATR inhibitors. Science.

[227] Robinson N.J., Miyagi M., Scarborough J.A., Scott J.G., Taylor D.J., Schiemann W.P. (2021). SLX4IP promotes RAP1 SUMOylation by PIAS1 to coordinate telomere maintenance through NF-kappaB and Notch signaling. Sci. Signal.

